# An Occupational Safety and Health Perspective on Human in Control and AI

**DOI:** 10.3389/frai.2022.868382

**Published:** 2022-07-06

**Authors:** Susanne Niehaus, Matthias Hartwig, Patricia H. Rosen, Sascha Wischniewski

**Affiliations:** Unit Human Factors, Ergonomics, Federal Institute of Occupational Health and Safety (BAuA), Dortmund, Germany

**Keywords:** human in control, AI-based systems, occupational safety and health (OSH), human factors, robotic systems, ICT

## Abstract

The continuous and rapid development of AI-based systems comes along with an increase in automation of tasks and, therewith, a qualitative shift in opportunities and challenges for occupational safety and health. A fundamental aspect of humane working conditions is the ability to exert influence over different aspects of one's own work. Consequently, stakeholders contribute to the prospect of maintaining the workers' autonomy albeit increasing automation and summarize this aspiration with the human in control principle. Job control has been part of multiple theories and models within the field of occupational psychology. However, most of the models do not include specific technical considerations nor focus on task but rather on job level. That is, they are possibly not able to fully explain specific changes regarding the digitalization of tasks. According to the results of a large-scale study on German workers (DiWaBe), this seems to be the case to some extend: the influence of varying degrees of automation, moderated by perceived autonomy, on workers' wellbeing was not consistent. However, automation is a double-edged sword: on a high level, it can be reversely related to the workers' job control while highly autonomous and reliable systems can also create opportunities for more flexible, impactful and diverse working tasks. Consequently, automation can foster and decrease the factor of job control. Models about the optimal level of automation aim to give guidelines on how the former can be achieved. The results of the DiWaBe study indicate that automation in occupational practice does not always happen in line with these models. Instead, a substantial part of automation happens at the decision-making level, while executive actions remain with the human. From an occupational safety and health perspective, it is therefore crucial to closely monitor and anticipate the implementation of AI in working systems. Constellations where employees are too controlled by technology and are left with a high degree of demands and very limited resources should be avoided. Instead, it would be favorable to use AI as an assistance tool for the employees, helping them to gather and process information and assisting them in decision-making.

## Introduction

Due to digitalization, jobs and working tasks are continuously changing. The development of recent technologies, such as artificial intelligence (AI) or advanced robotics has established new possibilities for task automation and revived the debate on work-related psychosocial and organizational aspects and on workers' safety and health. Amongst other things, these new technologies have the capability to fundamentally change the workers' perceived level of autonomy (Arntz et al., 2020; Wang et al., 2020; Fréour et al., 2021). The reason lies within a key feature of modern AI, its ability to operate and adapt without human intervention, in other words, autonomously while the human is left with supervisory or ancillary activities. It should be noted that automation is not equivalent to functioning autonomously. AI is used to automate functions to a certain degree, often following pre-programmed rules which makes it necessary for an operator to be present and to perform certain tasks before or after. Only if the human is not required for input or guidance, the system is seen as autonomous. In most cases, a high level of automation is reversely related to the workers' freedom in how to perform a certain task and how or what to use while completely autonomous and reliable systems can create opportunities for more flexible, impactful and diverse working tasks (Parasuraman et al., 2000; Moore, 2019; Rosen et al., 2022). Therewith, AI-based systems hold the potential to a qualitative shift in opportunities and challenges for occupational safety and health (OSH). AI-based systems are not entirely new, however their availability, complexity, performance and scope of capabilities have been extremely enlarged by the increase in computational power within the last years (Hämäläinen et al., 2018). Definitions of AI have therefore been constantly changing as they are adapting to technological advances. The term has been defined in numerous ways and a universal definition of an AI-based system is not agreed upon. However, it can be helpful to look at the definitions of major stakeholders like the OECD (2019) and the European Commission (2021).

The OECD (2019) defines AI-based systems as follows:

[…]a machine-based system that is capable of influencing the environment by making recommendations, predictions or decisions for a given set of objectives. It uses machine and/or human-based inputs/data to: (i) perceive environments; (ii) abstract these perceptions into models; and (iii) interpret the models to formulate options for outcomes. AI systems are designed to operate with varying levels of autonomy. (OECD, 2019)

An expert group on artificial intelligence set up by the European Commission, presents the following definition:

“Artificial intelligence (AI) refers to systems that display intelligent behaviour by analysing their environment and taking actions—with some degree of autonomy—to achieve specific goals. AI-based systems can be purely software-based, acting in the virtual world (e. g., voice assistants, image analysis software, search engines, speech and face recognition systems) or AI can be embedded in hardware devices (e. g. advanced robots, autonomous cars, drones or Internet of Things applications).” (EU, 2019)

Both concepts have in common that they include the varying degrees of autonomy in AI-based systems as well as their ability to perceive their environments in some way, analyze the information and act in response with different degrees of autonomy. It is therefore known that interacting with these systems often includes humans to rely on the machine's complex information-processing functions like sensory processing, information storage and analysis capabilities for, amongst others, decision-making (McCormick and Sanders, 1982; Kaber and Endsley, 1997; Parasuraman et al., 2000). With this, the implementation of AI can not only shift tasks from manual to more cognitive tasks, it also creates the risk of removing “operators from direct process control” and imposing high monitoring workload (Kaber et al., 2009). Moreover, highly automated systems have implemented algorithms that enable them to adapt, learn and function autonomously. This might curtail the workers' freedom as these systems have a low level of transparency that lowers the understandability and predictability of their actions. Therefore, it is difficult, if not impossible for the worker to understand how decisions are made or how to resist them (Ajunwa, 2020). Different stakeholders named both the principle of transparency and the **principle of the human being in control or preserving workers**' **autonomy** as the most important aspects when designing AI-based systems. The latter (human in control/preserving autonomy) is addressed within the principles presented by the EU Commission, ETUC, ETUI as well as in the European Social Partners Framework Agreement on Digitalization. This agreement is a shared commitment of the contributing partners “to optimize the benefits and deal with the challenges of digitalization in the world of work” (ETUC, 2020). It includes a chapter especially dedicated to “Artificial Intelligence (AI) and guaranteeing the human in control principle.” The principle is related to OSH, especially to psychosocial risks, as a low level of autonomy can have negative effects on motivation, job satisfaction as well as on the employees' health and performance (Dwyer and Ganster, 1991; Melamed et al., 1995; Spector, 1998; Inoue et al., 2010; Rosen and Wischniewski, 2019; Arntz et al., 2020). The agreement demands the guaranteed control of humans over machines and AI in the workplace.

Our research questions in this study are twofold:

1. What Models Are Currently Employed to Estimate the Possible Role and Impact of Automation of Decisions on a Human-Centred Design Work?
2. What Is the Link Between Automation of Decisions at Work on Psychosocial Working Conditions of Employees? Two Answer These Research Questions, Theories and Models on Human in Control Are Presented Together With Recent Scientific Literature That Depicts Possible Effects of Digitalization and Automation on Workers' Wellbeing. Furthermore, the Results of the German Survey “Digitalization and Change in Employment (DiWaBe)” Will be Presented. The Study Intended Among Other Aspects to Investigate how Workers Are Impacted by Automation Technologies Like ICT or Production Machines That Give Instructions to the Worker and With This, Possibly Decrease Worker Control. These Systems per se Are not Purely AI-Based, However the Ability to Give Instructions Is Already an Advanced Function Which can be Even Extended by the use of AI. This Section Will be Followed by a Discussion About the Applicability of Presented Theories and Models on AI-Based Systems and Concluding Remarks on the Design of These Systems From a Human Factors Perspective.

## Theories and Models on Human in Control

The term “human in control” can be viewed as a certain level of autonomy that a worker has, for example, about decision-making, timing control and used methods during a working task. Therefore, it is closely linked to the psychosocial working condition of job control that comprises different aspects like timing or method control or decision latitude that consists of decision authority and skill discretion. Another term that closely relates to the same concept is referred to as job autonomy or task autonomy. Within scientific literature, these terms are often used interchangeably albeit one might argue that there are slightly different nuances to them. However, the combining element is to exert influence over different aspects of one's own work (Semmer, 1990). The idea of this fundamental workplace resource can also be found in the human in control principle. The human in control principle, as was recently argued by the European Trade Union Confederation (ETUC), is one of the most important measures when designing artificial intelligence (AI) or machine learning systems in order to create the opportunity for good working conditions despite increasing levels of automation (ETUC, 2020). Research in the field of occupational psychology shows that in particular low levels of job control and a small extent of task variability can have negative effects on motivation, job satisfaction as well as on the employees' health and performance (Dwyer and Ganster, 1991; Melamed et al., 1995; Spector, 1998; Rosen and Wischniewski, 2019; Arntz et al., 2020). Job control or autonomy is therefore known as a fundamental task characteristic and has the potential to enhance job performance and increase motivation (Gagné et al., 1997; Morgeson et al., 2005; Ter Hoeven et al., 2016). However, technological developments and innovations, such as artificial intelligence, give rise to new possibilities for task automation that have the capability to fundamentally change the workers' perceived level of autonomy (Arntz et al., 2020; Wang et al., 2020; Fréour et al., 2021). Overall, it has been shown that automation can either benefit or decrement workers' performance and wellbeing, depending on the task itself, the organizational structure/environment, design implementation and the machine's level of autonomy (Wiener and Curry, 1980; Kaber and Endsley, 1997, 2004; Parasuraman et al., 2000; Arntz et al., 2020). Negative influences occur when automated systems have a low level of transparency and make humans rely on AI-based algorithms as they perform all complex information-processing functions. This can lead to out-of-the-loop (OOTL) performances that have been proven to be accompanied by negative effects such as vigilance decrements, complacency, loss of situation awareness and skill decay (Wiener and Curry, 1980; Kaber and Endsley, 1997, 2004; Endsley and Kaber, 1999; Gouraud et al., 2017). Nevertheless, the automation of routine tasks and the implementation of artificial intelligence can also decrease redundancy, improve safety conditions and create opportunities for more stimulating, challenging, and impactful working tasks (Moore, 2019; Rosen et al., 2022). In order to find modes in which the distributions of functions to a human or machine will increase performance while preventing the mentioned negative consequences, research has focused on presenting theories on levels of automation (LOAs) and degrees of automation (DOAs) (Kaber and Endsley, 1997; Parasuraman et al., 2000; Kaber et al., 2009; Wickens et al., 2010). Accordingly, the goal when designing AI is to develop methods for a human-machine interaction in which humans are not only in the loop but are enabled to be in control when making decision while aided by technology which goes in line with the human in control principle.

The following paragraphs will describe established models and theories that focus on the psychosocial working condition of job control as well as on degrees and levels of automation (DOAs; LOAs). Depending, selected scientific literature will be presented that depict the effects of automation and digitalization on workers' health, performance and sense of control over the working situation.

### The Scope of Activity by Ulich

Ulich (2005) presents a theory on the effect of working conditions on people and focusses on job autonomy or job control. His theory is based on the assumption that job autonomy is a multidimensional construct and is comprised of three components that are equally important for human-centered and health-maintaining design of work: scope of action (“Handlungsspielraum”), the scope of variability/creativity (“Gestaltungsspielraum”) and decision latitude (“Entscheidungsspielraum”). Ulich describes the **scope of action** as the degrees of freedom in the execution and temporal organization of work actions (flexibility). He further differentiates between the objective and subjective job autonomy. The former is described as the actual available choices while Ulich understands the latter as the *perceived* options of action. The **scope of variability/creativity** is described by Ulich as the extent to which the worker has the opportunity to independently design their work and procedures. The amount of variability of partial actions and partial activities thus creates differences in the present scope of creativity. **Decision latitude** is the third component in Ulich's theoretical framework and describes the extent of an employee's decision-making authority and autonomy to independently determine and delimit working tasks. According to Ulich, a higher occurrence of each of these components has a positive impact on the workers' health.

### Job Characteristic Model

**Figure 1:**
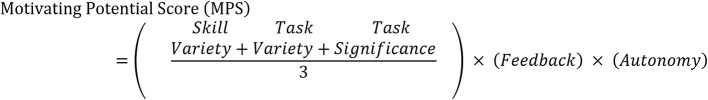
Equation on Job Motivation.

While Ulich structured and systematized a multidimensional construct to make general assumptions on the effect of job control or autonomy on employees health, the job characteristics model by Hackman and Oldham (1975) focusses on determinants of intrinsic job motivation. Hackman and Oldham provide a theoretical explanation for the level of intrinsic motivation, depending on work characteristics and workers' mental states. The core work characteristics in their model include skill variety, task variety, task significance, autonomy and feedback. These lead to the experience of meaningfulness at work, of responsibility for work outcomes and the knowledge of work results. They particularly emphasize the concept of autonomy and postulate that the possibility to influence the course of the work activity or of decision-making is a key factor for intrinsic work motivation. Moreover, their equation (see Figure 1) presupposes the presence of autonomy for any amount of work motivation, measured by the motivation potential score (MPS). Similar to the theory by Ulich (2005), Hackman and Oldham (1975) postulate a positive linear relationship between all five core characteristics and the outcome variables. In their model, **skill variety** is described as the extent to which a job requires different activities to carry out the work involving a number of different skills and talents of the person. **Task identity** is defined as to what extent a job is holistic and produces identifiable work results. **Task significance** represents the degree to which the activity that is carried out has a substantial impact on the life or work of other people. The core characteristic **autonomy** is specified as the scope of freedom, independence and discretion the human has regarding scheduling and procedures. The model supposes a linear relationship between autonomy and motivation as the authors claim that the more freedom, the stronger the employee's motivation will be. The last factor to influence work motivation and satisfaction in the equation by Hackman and Oldham is **feedback**, which is described as the extent to which an employee will get clear and direct information about their task performance. Besides autonomy, feedback is the only other factor that must be present in order to yield any motivation (see Figure 1).

### Job-Demand-Control Model (JDC)

**Figure 2:**
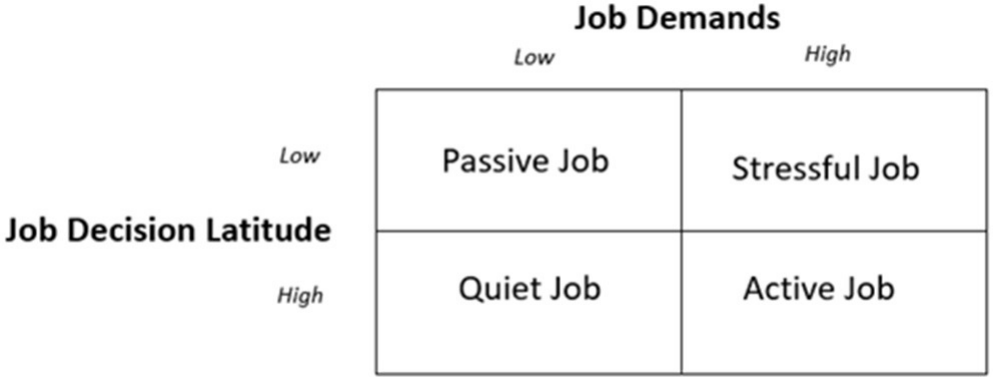
Job-Demand-Control Model by Karasek (1979).

The Job-Demand-Control (JDC) model by Karasek (1979) and Karasek and Theorell (1990) focusses on the stress potential of different jobs. According to Karasek (1979), the perception of acute strain and stress in working situations depends on two dimensions, namely job demands and decision latitude. Hereby, the work-specific requirements account for the extent of perceived job demands while decision latitude is explained as the degree of task variety and decision autonomy. Karasek and Theorell (1990) understand control, that is, a high level of decision autonomy and task variety, as a requirement for good working conditions, which is in line with the before mentioned models. However, to characterize types of jobs with different stress potential, they also rely on the existing job demands. As a result, four possible types are postulated: the quiet job (low work requirements and large scope of decision latitude), the passive jobs (both dimensions are low), the stressful job (high work requirements with low levels of task variety or decision latitude) and the active job (both dimensions are high). The latter is seen as the job with optimal stress and as overall health promoting while the stressful and passive job causes health risks, over- or underload as well as a decline in abilities and activities (Karasek, 1979). Although the quiet job is not believed to be detrimental to the person's stress level, Karasek (1979) assumes that people will not add to their competency on the job and generally in life if the job demands are not matched with the skill or control they experience. Therefore, he supposes that more demanding jobs, which are accompanied by a high level of decision latitude or job control are the most desirable. An overview of the relationships postulated by the JDC model are shown in Figure 2.

### Job-Demand-Resources Model

A broader scope of work-related stressors and resources compared to the Job-Demand-Control model is incorporated by the Job-Demand-Resources model by Demerouti et al. (2001). It does not only focus on job control, but includes a number of work-related demands and resources that can influence the development of motivation and occupational stress. Demerouti et al. (2001) include a wide range of working conditions that they classify as either resources that help achieving work goals, reduce job stressors and stimulate personal development, or as demands, which are factors that require sustained effort. The former category includes, for example, job control, social support, and task variety. The latter contains factors that increase the possibility for disengagement and exhaustion such as emotional pressure, workload, and time constraints. The Job-Demand-Resources model assumes that an accumulation of demands, without the worker having enough personal or environmental resources, leads to a health impairment process. However, this entails that strengthening the workers' resources can alleviate perceived stressors and both sides are always interacting with each other. Demerouti (2020) proposes that it is possible to turn automation into a resource rather than a stressor for workers when technology is designed to support decision autonomy and helping the worker with highly complex decisions while taking over redundant and heavy tasks. Moreover, Demerouti (2020) points out the importance of supporting employees through the implementation of new technological systems to diminish newly occurring demands such as changes in work routine or the acquirement of new knowledge. With this, Demerouti's model is highly applicable in today's digitalized world of work.

### Vitamin-Model

**Figure 3:**
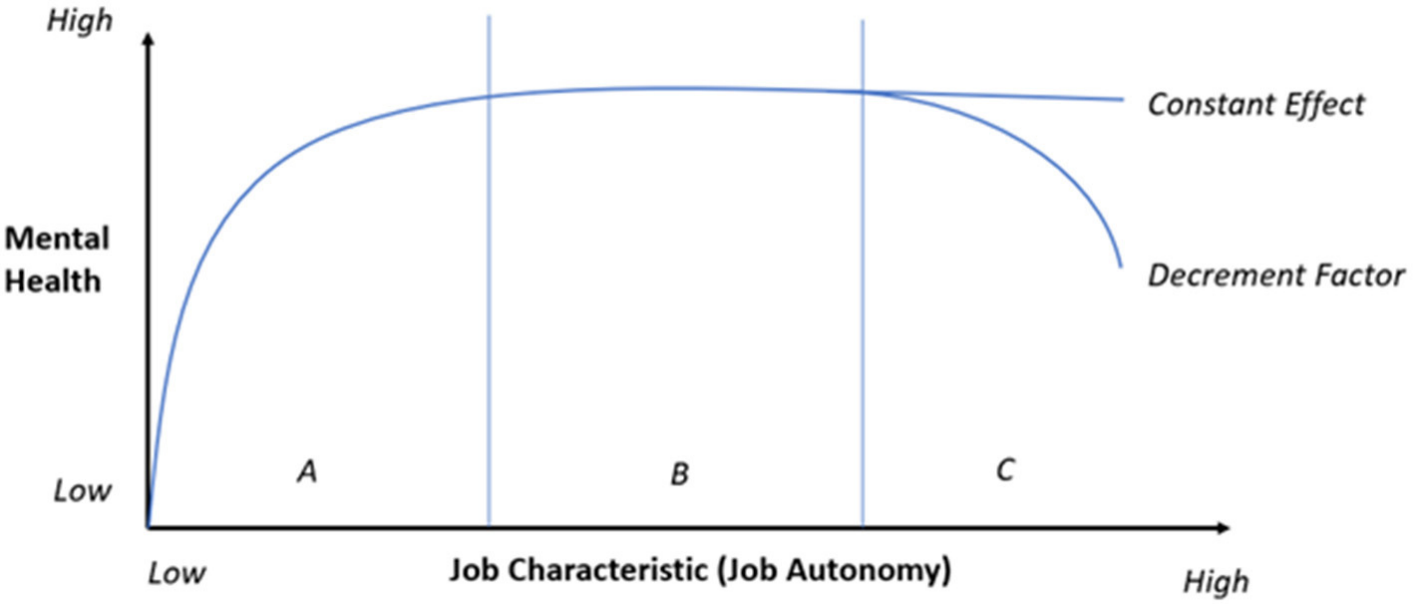
Vitamin-Model. Positive effect of increasing autonomy **(A)**, plateau **(B)** and additional decrement **(C)**.

In contrast to the other presented theories, the vitamin model by Warr (1987) differentiates between constant and decrement factors. That is, for some factors, Warr assumes not a linear but an inverted u-shaped or a saturation curve-relationship between their extent and mental health. Warr counts physical security, the availability of financial resources and a social position that favors self-esteem and recognition by others as constant effect factors. These can have a negative influence on workers' health if their occurrence is low but do not impact the worker positively if they exceed a sufficient level. That is, they hit a plateau (Warr, 1987). To the decrement factors, Warr denotes job control, the possibility of social contacts, the opportunity to develop and apply one's own skills, task variety (chance for new experiences) and the predictability and transparency of events. According to Warr, these follow an inverted u-shape. That is, the model predicts a negative impact on the worker's health if, for example, the level of job control is too low *or too high* (see Figure 3). However, the model lacks the specification of an optimal extent of autonomy. This uncertainty about the optimal level of autonomy is also present in theoretical considerations on LOAs as well. Nevertheless, most often a medium LOA is assumed to be beneficial which is more congruent with the assumption of a u-shaped relationship than a linear one.

### From Psychological Models to Theories About Optimal Automation

As described before, automation refers to a set of functions that are performed automatically by technology. With a low degree of automation, the worker has overall control of the technology while transferring some of it, over a specific function, to the machine. However, automation can, in its varying degrees, lead to less interaction of the worker with the working task, leaving her with ancillary activities or supervisory control. With this, automation can have positive and negative effects on the workers' performance and wellbeing. According to the aforementioned models, the perceived level of control or job autonomy takes over a mediating role in this interplay. As stated, the continuous automation of tasks has the power to change the employees' level of job control, that is, possibilities to decide upon task variety, used methods and timing (Arntz et al., 2020; Wang et al., 2020; Fréour et al., 2021). Researchers have therefore tried to give guidelines on how automation can increase job performances and satisfaction instead of fostering skill decay, complacency, workload or OOTL performances. In the following, two fundamental models on LOAs will be described that have been the basis for most of the current research.

### Ten-Level Model by Kaber and Endsley

The first model concerned with the optimal level of automation that will be described here, was put forth by Kaber and Endsley (1997). They developed a ten-level model of automation, ranging from manual control (Level 1) to full automation (Level 10) which gives a detailed description of who should be in charge of what function during the interaction. They present 10 levels of automation (LOAs) as well as ways in which the human and the machine could operate on different intermediate levels of automation that included shared control over the situation in order to identify scenarios beneficial for the human's situation awareness and for reducing workload (Kaber et al., 2009). They found that performance was best under low-intermediate levels of automation while higher levels of automation decreased the ability to recover from, and perform during, automation failures while manual control had a negative impact on performance and workload. One of the optimal scenarios included shared monitoring, planning and option selection with the final power of decision resting with the human. That is, Kaber and Endsley propose a medium LOA for positive effects on performance, situational awareness, operational safety and workload.

### Theoretical Guideline on Automation by Parasuraman et al.

The model by Parasuraman et al. (2000) about types and levels of automation gives a detailed theoretical guideline to what kind of task should be automated in order to decrease mental workload and skill decay while not encouraging loss of vigilance, situation awareness or complacency. He supposes that the effect that automation has on workers depends on the kind of task that is automated as well as on the level of automation. Therefore, he established a model that systematically shows which tasks should be automated, and to what extent. With this, it is intended to assign the control between a human operator and machine in an optimal way. Parasuraman et al. differentiate four types of automation (acquisition, analysis, decision, and action) and a continuum of automation from high to low. The level of automation is then evaluated by the degree to which it influences certain human performance areas such as mental workload, complacency, reduced situation awareness and skill degradation which he describes as “potential costs” (Parasuraman et al., 2000). According to Parasuraman et al., OOTL performance problems arise if these costs are too high. The level can be adjusted in an iterative manner before secondary evaluative criteria are applied. These include automation reliability and costs of decision or action outcomes. This process is repeated for all four types of automation. Parasuraman et al. also address the question, under which circumstances decision-making should be automated and in what scenarios it would not be suitable. As mentioned in previous paragraphs, a low decision latitude influences job satisfaction, motivation and, therewith, mental health, negatively. According to Parasuraman et al., this only occurs if the wrong tasks are automatized or if the level of automation is picked too high. Nevertheless, he notes that high-level automation and even full automation can be considered for decision-making if human operators are not required to intervene or take control under system failure as well as if they have time to respond (Parasuraman et al., 2000). Otherwise, high levels of automation would not be suitable since it would have a negative impact on mental workload, situation awareness and human performance.

Both theories on LOAs propose that assisting technologies that leave the action selection and the protocol development to humans and thus give workers control over the execution of tasks are more appropriate for tasks of great expertise while simple and redundant tasks can be performed by completely autonomous systems without negatively affecting the workers' autonomy.

### Exemplary Studies on Automation and Human in Control

The described models show the importance of the level of autonomy, job control and decision-making for workers. Consequently, theories on LOA try to provide a framework to include an optimal level of these parameters within the changing nature of work. However, there is no consensus on the effects of automation on workers as the automation of tasks can be either perceived as a stressor (e.g., restriction of autonomy/control) or as a resource (e.g., ability expansion), depending on the task itself, the environment and the level of automation (Parasuraman et al., 2000; Robelski, 2016; Demerouti, 2020; Wang et al., 2020). Demerouti (2020), for example, proposes that automation can be a resource if heavy and redundant tasks are taken over by the technology while employees are assisted in dealing with their changing work environment. A changing work environment could for example refer to the implementation of new AI-based technologies and the increase of information processing, while being supported in decision-making, learning and personal development. Following this section, a large-scale study about the effects of automation on German workers will be described in detail.

Fréour et al. (2021) interviewed 3 types of employees (i.e., experts, managers, users) from an organization which has started a digitalization process and conducted a study on changing work characteristics. They assumed that the more instructions humans get from machines, the more their perceived level of autonomy diminishes. As shown in the review by Wang et al. (2020) a number of studies conducted in laboratory setting indicate a negative effect of ICT use on time pressure and workload. However, Fréour et al. (2021) showed that the workers' autonomy was not reduced when digital technologies executed repetitive tasks (Fréour et al., 2021). Moreover, their results indicated that technology that takes over action selection on tasks that require low human control and expertise enhances the workers' perceived level of autonomy by accomplishing less interesting tasks and giving the workers more time on tasks with added value. This is in line with the model by Parasuraman et al. who suppose that different situations can be more or less suitable for automation. Human autonomy should be favored if a large extent of expertise or variability is needed whereas automation is recommended for repetitive and predictable tasks or situations in which a quick reaction time is crucial. Wickens et al. (2010) conducted a meta-analysis of 18 experiments on the effect of varying LOA and included performance and workload as an outcome parameter. Again, automating redundant work had positive effects such as performance and decreased workload (if the system functioned properly). The ameliorating effects of both studies on working conditions find a theoretical basis in the models of Ulich (2005), Hackman and Oldham (1975) as well as Karasek (1979) and Demerouti et al. (2001) since task variability, significance, and (decision) autonomy were increased through the higher LOA, resulting in an overall better condition. However, there are scenarios in which the automation of tasks increases mental workload and has a detrimental effect on situational awareness, the feeling of control as well as task variability (Kaber and Endsley, 1997; Endsley and Kaber, 1999; Weyer et al., 2015). This is often the case when a high LOA is implemented and the human is left with supervisory control over the system and only is expected to take control if the system fails (Wiener and Curry, 1980; Kaber and Endsley, 1997; Weyer et al., 2015; Gouraud et al., 2017). Parasuraman et al. (2000) mentioned that the reliability of the system is a key factor when it comes to lower the stress for the worker and impede overreliance on technology. A study about smart cars showed correspondingly that a higher level of automation increases satisfaction, but only if the malfunctions were low (Weyer et al., 2015). Other areas in which taking away human control can have positive effects are controlling the workers through occupational accident analysis, decision support systems or video surveillance for anomaly detection to prevent the occurrence of accidents and increase the workers' safety. Nevertheless, a study by Bader and Kaiser (2017) showed that ICT can foster the workers' feeling of being under control/surveillance and therewith curtail their freedom on working methods, scheduling of tasks and overall decision-making. Another negative consequence of these highly automated environments is the workers' loss of manual skills and the feeling to not be in control anymore (Berberian et al., 2012). The results by Berberian et al. (2012) suggest that the feeling of control is enlarged when action alternatives can be generated and selected as well as through greater involvement preceding an automated function. These conflicting arguments show the importance of a human-centered perspective when implementing AI or automating functions as well as the employees' opportunity to feel in control.

Overall, the studies suggest that the implementation of different LOAs can influence the employees' job autonomy and their sense of control. Although there are no clear results on the effect of specific LOAs on mental health, they do affect task variety and decision latitude as well as method and timing control, which in turn have been shown to influence the worker's perceived stress-level and overall health. Most findings suggest that automation is beneficial for redundant tasks that do not require the human to intervene in cases of system failure or if the takeover of manual control is easy. Negative effects occur if humans are left with supervisory control and redundant or ancillary activities. The “Ten-Level-Model” and the “Model for Types and Levels of Human Interaction with Automation” propose a medium level of automation for most tasks but clarify that multiple factors play into the decision on which tasks should be automated in order to influence performance and the worker's wellbeing positively. A key aspect of automation is the level of transparency that humans are able to experience when working with automated systems. Moreover, a high reliability should be given, as well as the possibility for the worker to take back control. In order to follow the human in control principle, it is necessary to take a human-centered approach and balance the degree of the system's autonomy with the level of desired control.

## Results of Digitalization and Change in Employment (DIWABE) Survey

The described models gave theoretical considerations on how much autonomy and job control are beneficial for the workers' wellbeing while the theories on LOAs and presented laboratory studies indicate that automation in itself influences the perceived level of human control and autonomy. However, until this day, studies on the actual situation in workplaces regarding the increasing automation and subsequent effects on task characteristics and the employees' wellbeing are rare. To fill this gap, the next paragraphs will describe in detail specific results of the German survey “Digitalization and Change in Employment (DiWaBe).” In this survey more than 8,000 employees answered questions on their working environment and conditions in order to find out how workers are impacted by automation technologies like ICT or machines. Of special interest are systems that give instructions to the workers and possibly reduce perceived job control. Moreover, the study assesses the current relation between decisions made by technologies, working conditions and mental health. The following paragraphs will include a short description of the survey and the results regarding the impact of technology in control.

The DiWaBe survey was jointly designed by the Federal Institute for Occupational Safety and Health (BAuA), the Federal Institute for Vocational Education and Training (BIBB), the Institute for Employment Research (IAB) and the Leibniz Centre for European Economic Research (ZEW) in 2019. The survey was conducted *via* telephone and included more than 8,000 employees from about 2,000 different German companies. These companies had already participated in a representative company survey (IAB-ZEW-Working World 4.0) in 2016 as a random sample stratified by region, company size and sector. Based on the population of all employees in these companies, participants in the DiWaBe study were also selected as a random sample stratified by age, gender and education level (for details, see Arntz et al., 2020). The questionnaire was specifically designed for the survey, including a differentiated assessment of working technologies, split up in the categories information and communication technologies (ICT) and machines/tools, which creates a unique data set. It also includes a wide array of questions regarding physical and psychological working conditions in form of stressors and resources, some of them oriented toward items in the Copenhagen psychosocial questionnaire (COPSOQ, see Kristensen et al., 2005) for comparability.

### Sampling and Data Preparation

The overall response rate is 16.43%, and the distribution of the interviews deviates relatively clearly from the distribution of the gross sample. It is particularly noticeable that the utilization rates of the education group high are (as expected) significantly higher than those of the other two education groups (low and med), which was later corrected *via* weighting of the data. For a detailed description of sampling and composition, see also Arntz et al. (2020). The gathered data was subsequently compared with administrative data and weighted by the variables mentioned above in order to be as representative as possible of the private sector in Germany. The individual weights were trimmed at the 95th percentile so that possible outliers would not have too much influence, possibly distorting the data. For the present analysis, the sample was restricted to currently employed individuals up to the age of 65 years (current age of retirement in Germany) with valid information on the main variables included. Moreover, persons with 200 or more days absent from work due to illness within the last 12 month were excluded because of potentially distorted answers after the prolonged absence. Table 1 shows the resulting sample.

**Table 1:** Sample description.

**Sample**	**%**	***n***
Total		6,153
Female	46.5	2,861
Age: 18–34	16.0	982
Age: 35–49	38.6	2,378
Age: ≥50	45.4	2,794
Qualification: No degree	6.5	399
Qualification: Apprenticeship/vocational	48.3	2,972
Qualification: Meister/Technician	14.3	881
Qualification: University degree	30.7	1,894
Working with ICT (at least rarely)	90.8	5,590
Working with machines (at least rarely)	49.2	3,026

After assessing the technology use, the participants answered questions on how often technology makes decisions about their work process and gives instructions to the participant, addressing the automation of decision aspect of the Parasuraman model. The item wording was: “How often does it happen that the technology gives you instructions, e.g., about the next work step?” (1 = never, 5 = always). As work with ICT and machines differ substantially, the analysis was carried out separately for both technology classes.

Table 2 gives an overview on the mean of working instructions by technology for different sociodemographic groups. Regarding ICT, male participants report slightly more instructions by ICT than women. Among all groups, people aged 50 and over report a slightly higher level of instructions than the other age groups. Between the different qualification levels, there is a slight but continuous decrease in instructions through ICT as the qualification level increases. Employees in occupations with higher qualification requirements report, on average, fewer instructions than those with low qualification levels. Throughout the different occupational sectors, the most instructions through ICT are reported in the production manufacturing jobs. People in other economic service occupations report the second highest value.

**Table 2:** Sociodemographics.

	**Instructions by ICT**			**Instructions by machines**		
	**Mean**	**SD**	***n***	**Mean**	**SD**	***n***
Total	2.28	1.25	5,446	2.24	1.29	2,315
Gender: male	2.30	1.26	3,038	2.18	1.27	1,638
Gender: female	2.25	1.26	2,551	2.42	1.32	723
Age: 18–34	2.25	1.24	891	2.09	1.21	485
Age: 35–49	2.22	1.25	2,186	2.29	1.33	947
Age: 50–65	2.34	1.27	2,511	2.30	1.29	929
Qualification: no qualification	2.57	1.61	179	2.24	1.45	123
Qualification: apprenticeship/Vocational	2.37	1.33	2,608	2.23	1.32	1,205
Qualification: master craftsmen/technician	2.23	1.16	1,308	2.35	1.31	513
Qualification: university degree	2.11	1.11	1,350	2.15	1.14	473
Branch: manufacturing jobs	2.41	1.27	1,433	2.20	1.29	1,163
Branch: personal services	2.21	1.33	1,193	2.24	1.28	508
Branch: commercial company-related services	2.22	1.13	1,927	2.29	1.30	282
Branch: IT and scientific service professions	2.22	1.20	376	2.29	1.19	199
Branch: other economic services	2.30	1.50	515	2.40	1.38	161

In case of instructions given by machines, women report a slightly higher level on average than men. Among the different age groups, the lowest level of control by machines is seen in the group under 35 years of age. The other two groups report an almost identical mean. Surprisingly, a different picture emerges regarding the skill requirements for machines compared to ICT. The highest mean level of instructions by machines is reported by master craftsmen and technicians, the group with a rather higher level of qualification and typically associated with less standardized tasks. In terms of occupational sectors, people in other business services report the highest level of instruction by machines, while the other sectors are at a similar level.

To explore the potential impact of technology in control, linear regression in separate models was used to predict the impact of reported instruction by technology on several aspects of work intensity, job control and burnout indicators. These items are based on the Job-Demand-Resources model as key factors of potential stressors and beneficial resources at work. According to the Job-Demand-Control model by Karasek (1979) as well as the Job-Demand-Resources model by Demerouti et al. (2001), an unfavorable constellation of demands and low resources, especially in the long run, leads to a decrease in health associated variables. Methodologically, the use of parametric tests has advantages and disadvantages over non-parametric tests for likert scale-data, depending on the sample, the items and the research question. After weighing these factors, especially the sample size and the item design which does not include verbal gradations of the items, in this study we follow the argumentation of Norman (2010) and use linear regression as a robust parametric test method for calculation. Table 3 shows the regression coefficients, standard error and standardized regression coefficients beta for instructions by ICT (left) and instructions by machines (right).

**Table 3:** Linear regressions.

**Independent variable**	**Instructions by ICT**			**Instructions by machines**		
	**Regr. coeff**	**Std. error**	**Beta**	**Regr. coeff**	**Std. error**	**Beta**
**Dependent variable**						
**Work intensity**						
Physical stress	0.109	0.014	0.101***	0.027	0.020	0.027
Multitasking	−0.030	0.010	−0.039**	0.016	0.016	0.021
Interruptions	−0.008	0.011	−0.010	0.035	0.016	0.045***
Information overload	−0.003	0.010	−0.004	0.094	0.015	0.131***
**Job control**						
Organizing work	−0.133	0.012	−0.147***	−0.054	0.020	−0.055**
Working speed	−0.094	0.012	−0.101***	−0.090	0.021	−0.090***
Task approach	−0.118	0.013	−0.125***	−0.064	0.020	−0.068**
Amount of work	−0.057	0.013	−0.058***	−0.058	0.020	−0.058**
Repetition of working steps	0.138	0.010	0.179***	0.139	0.015	0.187***
**Burnout indicators**						
Physical exhaustion	0.022	0.012	0.025	0.090	0.018	0.101***
Emotional exhaustion	−0.015	0.012	−0.017	−0.031	0.018	−0.036
Feeling drained	0.016	0.012	0.017	0.064	0.019	0.071**

*2,341 < n < 5,592*.

*p < 0.05, **p < 0.01, ***p < 0.001.

### More Physical Stress With Instructions by ICT

**Figure 4:**
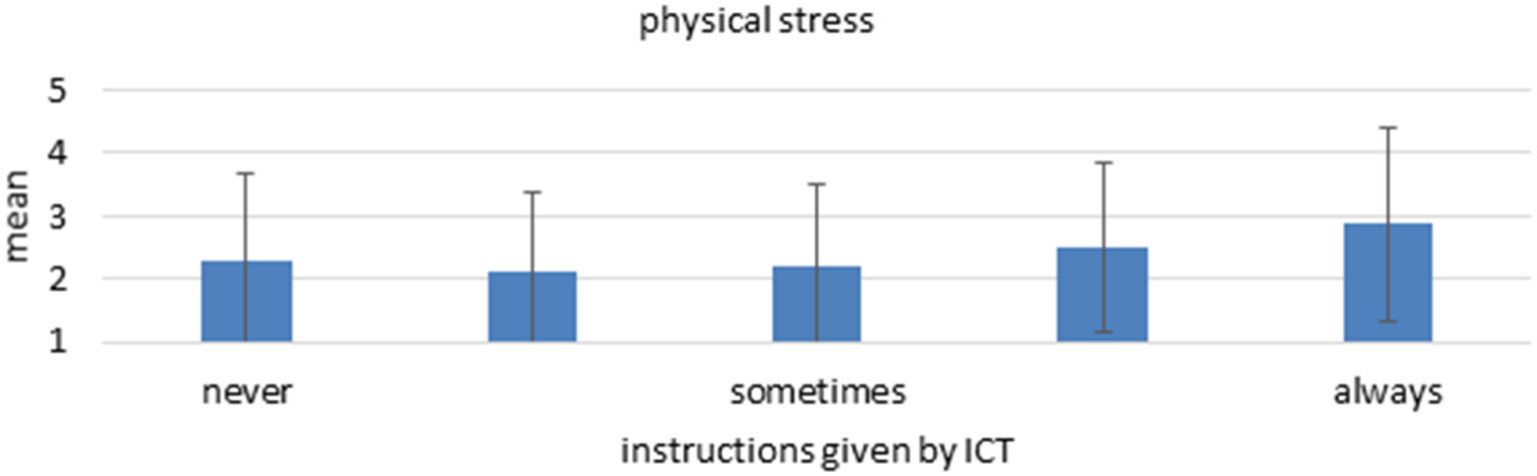
Mean and standard deviation of the item “physical stress” among the different “Instructions by ICT” groups. *n* = 5.586, linear regression coefficient beta = 0.101, *p* < 0.001.

**Figure 5:**
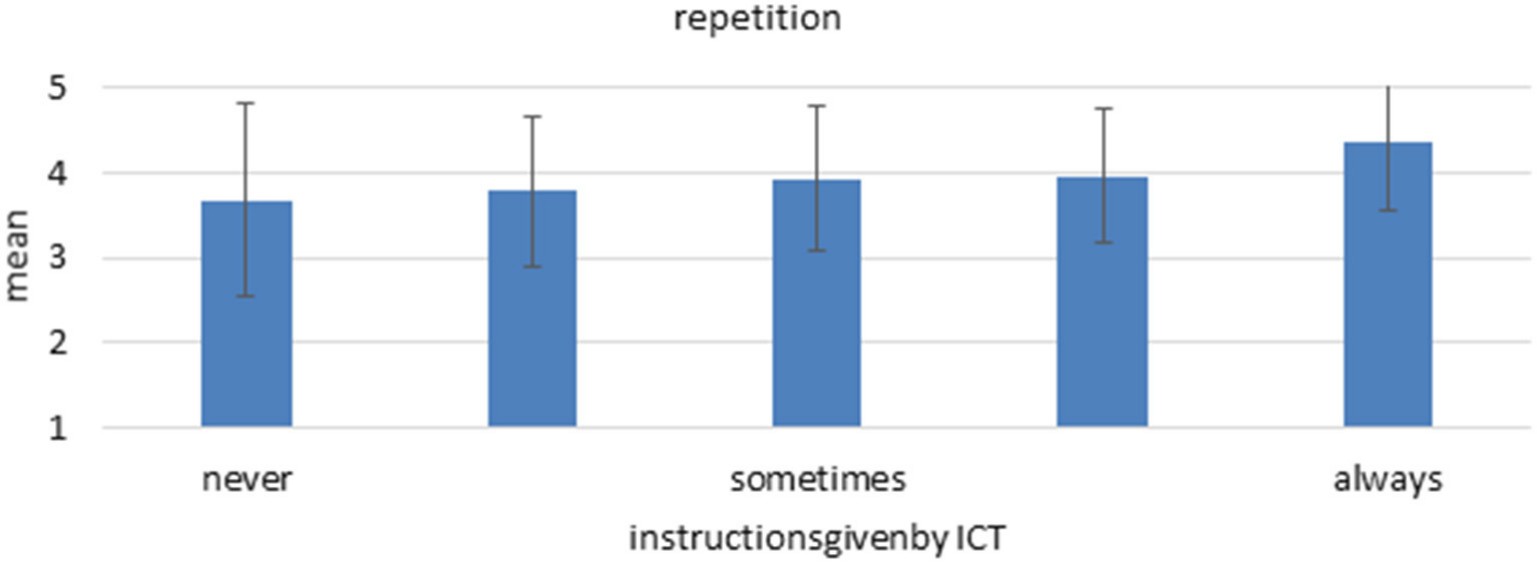
Mean and standard deviation of the item “repetition” among the different “instructions by ICT” groups. *n* = 5,587, linear regression coefficient beta = 0.179, *p* < 0.001.

The statistical models prove that instructions by ICT are a significant predictor for multiple aspects of work intensity as well as all facets of job control. More specifically, regarding work intensity, a higher degree of instructions by ICT is associated with more physical stress (Figure 4). Surprisingly, higher levels of instructions by ICT are also connected with mildly less multitasking, which might indicate that work is more standardized and closely supervised with less parallel subtasks when automated. This would indicate that a high LOA regarding decision-making is implemented which leaves the human with more focus on (physical) action. This interpretation would be in line with the results regarding job control. Here, instructions by ICT predict all facets and high levels are associated with less freedom in organizing one's work, influencing the working speed, the possibility of choosing between different task approaches and influencing the amount of work. The strongest relation exists for repetition of working steps, where higher levels of instructions by ICT predict a substantial higher level of repetition of working steps (Figure 5). Regarding mental health, however, no relation is found between the instructions by ICT and indicators of burnout. This goes against the assumptions of Ulich (2005), Karasek (1979), Hackman and Oldham (1975) or Warr (1987) since they all propose a negative influence of low levels of job control on mental health. However, the model by Demerouti et al. (2001) could provide an explanation for the missing link as it suggest that existing resources can alleviate the negative effects of stressors, such as low job control. Possibly, employees that work with ICT have more personal resources or better social or management support. The models show no significant predictions for interruptions and information overload.

The presented models on LOAs can only be applied partly to these results, as they focus on the technical implementation and are task specific. They assume that an increase in workload after task automation is an indicator for an incorrect choice of task or for a level of automation that is picked too high.

### More Information Overload With Instructions by Machines

**Figure 6:**
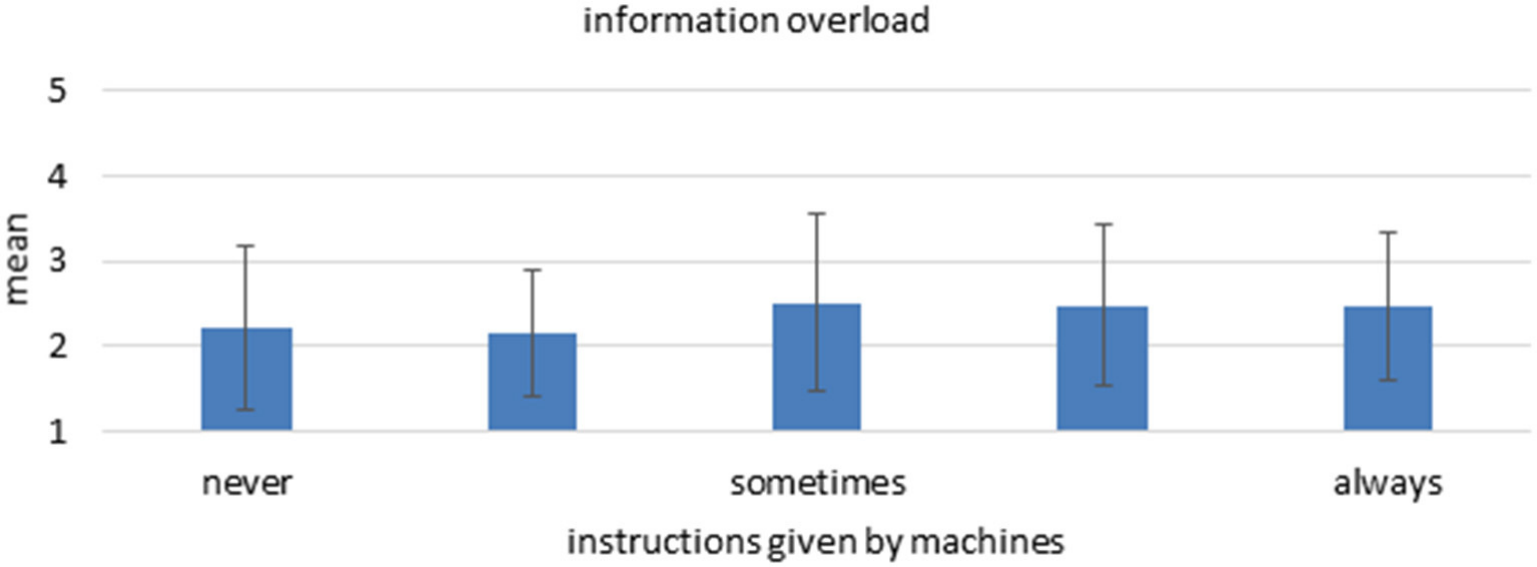
Mean and standard deviation of the item “information overload” among the different “instructions by machines” groups. *n* = 2,355, linear regression coefficient beta = 0.129, *p* < 0.001.

When predicting work intensity regarding varying amounts of instructions by machines, a different pattern emerges. Higher levels of instructions by machines are associated with significantly more interruptions and more information overload (Figure 6). There was no significant prediction for physical stress or multitasking, however. Again, theories on LOAs would argue that these results are an indicator for a wrongly chosen task to be automatized or a higher level of automation than would be necessary or beneficial.

**Figure 7:**
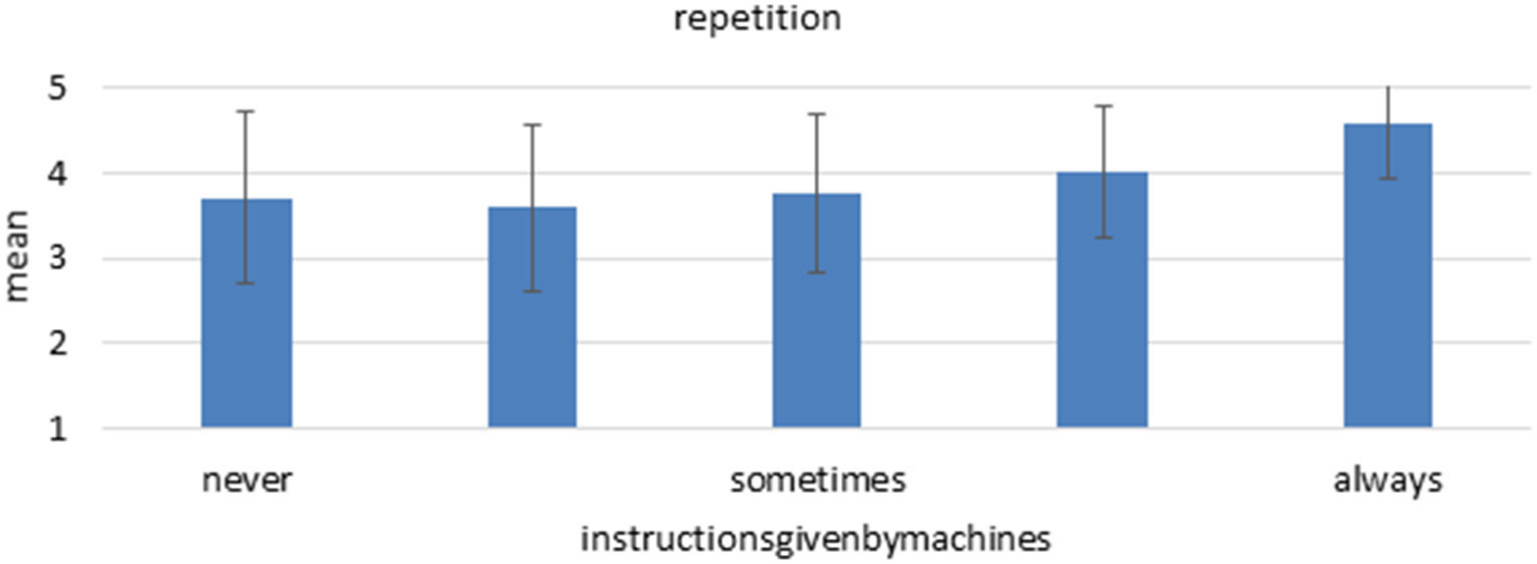
Mean and standard deviation of the item “repetition” among the different “instructions by machines.” *n* = 2,361, linear regression coefficient beta = 0.184, *p* < 0.001.

Regarding decision latitude and overall job control, results show an identical pattern between instructions by ICT and machines. Participants reporting more instructions by machines also report significantly less job control among all facets, although some predictions are weaker than those of instructions by ICT. The strongest relation is again found between instructions by machines and repetition, where more instructions are significantly associated with more repetition of working steps (Figure 7). Participants, who reported that they always receive instructions by machines, also reported usually executing the same subtask over and over again. It again highlights the assumptions in the Parasuraman model that while automation of *already* redundant tasks is beneficial for decision latitude and performance, automation of decision-making often does not lead to better working conditions.

**Figure 8:**
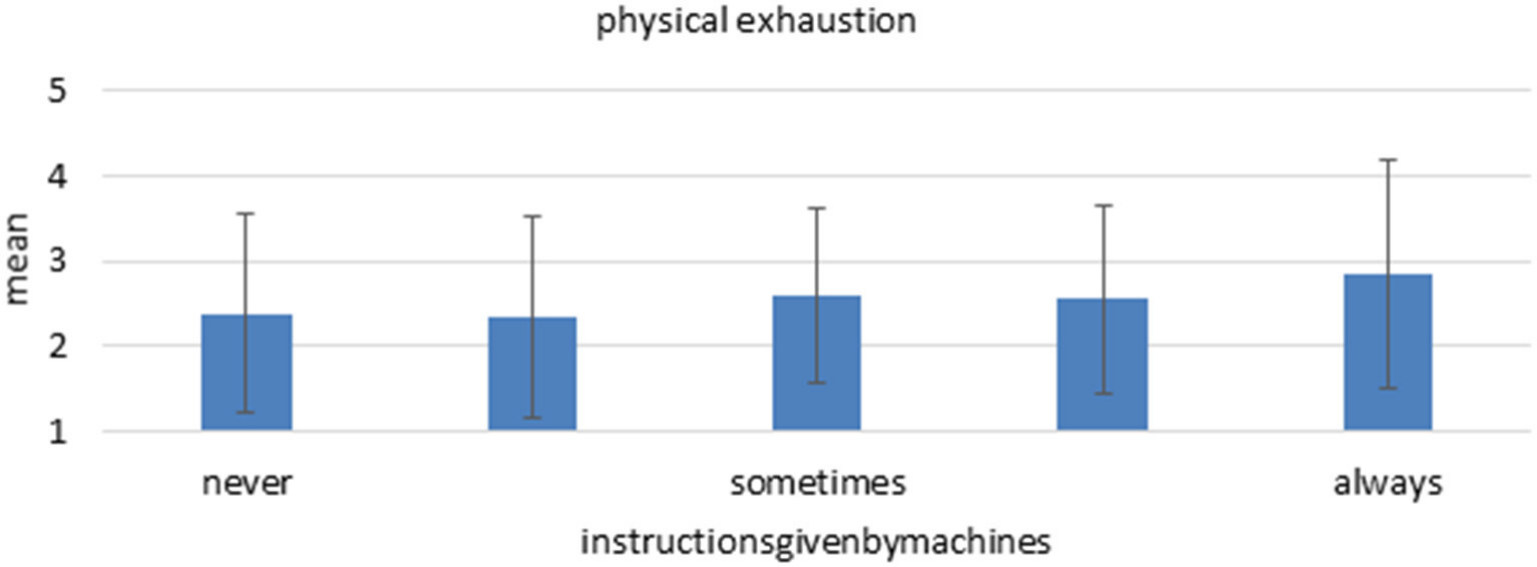
Mean and standard deviation of the item “physical exhaustion” among the different “instructions by machines.” *n* = 2,356, linear regression coefficient beta = 0.101, *p* < 0.001.

Instructions by machines proved also to predict two out of three burnout screening items. High level of instructions by machines were associated with more feeling of being drained and more physical exhaustion (Figure 8). As a higher level of instructions is correlated with job control, the shown negative impact on mental health is according to the presented models by Ulich (2005), Karasek (1979), Demerouti et al. (2001) and Hackman and Oldham (1975). The vitamin model by Warr (1987) predicts that too much control also can have negative effects, which can be partly seen in the present data. Furthermore, the authors of theories on LOAs founded their models on the fact that redundant working conditions and less decision autonomy has detrimental effects on workers. Therefore, the present results go in line with these theoretical considerations as well as with other studies in laboratory settings (Kaber and Endsley, 1997; Endsley and Kaber, 1999; Parasuraman et al., 2000; Weyer et al., 2015). However, according to Parasuraman et al. (2000) as well as Kaber and Endsley (1997), these negative effects occur only in case of weak technical reliability, wrong task selection or overly high level of automation.

In sum, the analysis provides a broad, explorative overview of the extent to which technologies currently exert control over employees' work activities and what working conditions go hand in hand with this. Overall, it shows that partial control of employees by automation technologies is already part of everyday working life. On average, the participants state that they receive “rarely” to “sometimes” instructions by technology about their next work steps. Older and lower-skilled employees are on average affected by instructions through technology slightly more often than other workers. With regard to the correlations of control through technology with relevant working conditions and indicators of mental health, a distinction must be made between the basic types of automation technologies. Different patterns can be found for ICT used for the automation of information-related tasks compared to technologies like production machines used for processing physical objects. While control by ICT systems is associated with a higher degree of physical stress and less multitasking, higher control by machines predicts more interruptions and an increase in information overload. In contrast, the correlations with job control such as facets freedom of action and degree of repetition in the activity as a whole, are similar. Here, more control by technology in both classes is associated with a decrease in job control. The results imply that control by technology does not only substitute control that was previously exercised by humans. Instead, control by technology seems either adding to existing control by superiors, or it seems to be associated with tighter instructions. It is striking that some facets of job design are already rated in the lower (autonomy and decision latitude) or upper (degree of repetition) range of the scale, and are rated even more extremely by participants that report more intensive decisions through technology.

These results are important, as a minimum level of autonomy is a relevant factor for the mental health of employees and represents a long-term risk for mental illness. Regarding control by machines, results indeed indicate a connection between the degree of control and screening facets of burnout symptoms, where more instructions by machines are associated with more physical exhaustion and more feelings of being drained. Overall, the results therefore point to a worsening of working conditions rather than an improvement, if more decisions are made by technology.

### Limitations and Evaluations of the Results

Regarding limitations of the study, several should be noted. Firstly, all empirical results are based on subjective data and are therefore prone to specific measurement error, for example due to biased personal perception of the situation. Within large samples, one can assume that random measurement error is somewhat nullified by large numbers. However, there is always a risk for a systematic error to distort the results, as employees are not randomly assigned to workplaces. Therefore, certain groups of employees might answer the questions systematically different than other subgroups due to confounding variables beyond the ones that were included as control variables, for example personal work motivation. There are approaches to handle this possible error by including employees wages and personal work histories (for example Böckerman et al., 2012). However, as the data set include thousands of employees, we did not use indirect methods to control for variables that were not directly in the data.

Additionally, the results cannot make a clear statement about the extent to which the correlations are due to decision-making by machines *per se*, or due to the specific design and implementation of technologies. Also, due to the exploratory nature of the analysis, a more in-depth investigation of individual subgroups of employees effects was not yet undertaken and will be subject of future research. For example, correlations might be substantially different for different subgroups regarding age, health, qualifications level, company, personality traits and so on. Due to these aspects, we rate the external validity of the data as medium. The big sample, careful sampling and weighting of the data leads to generally high global validity, limited by a non-randomized setup and subjective data. In addition, due to the high abstraction level and the therefore very heterogenic sample, individual results in a specific setting might differ substantially.

## Discussion

The digital transformation of work is apparent across all sectors and therewith, entails fundamental changes for the world of work and society. Multiple aspects of today's work, including task characteristics, work environment, health and safety can profit from digitalization and automation in terms of increased productivity, more creative freedom in organizing work and new job opportunities. However, this shift in digitalization can also pose risks and challenges for workers when they are not included in the process and changes are not anticipated correctly. Due to the extraordinary increase in computational power, AI-based systems get more available, complex and capable day by day and therefore hold the potential to qualitatively impact occupational safety and health. AI-based systems are built to automate certain tasks and are even able to work autonomously with little human control, which can be a threat to job autonomy. Theories and models from the field of occupational psychology have argued that a decrease in the factor of job control which involves the possibility to choose tasks, working methods and procedure as well as decision autonomy has detrimental effects on workers' wellbeing. Consequently, stakeholders contribute to the prospect of maintaining the workers' autonomy albeit increasing automation and summarize this aspiration with the human in control principle. The mentioned models agree on the fact that autonomy is a fundamental aspect of good working conditions and is crucial to ensure motivation, job satisfaction and mental health. However, the models are not AI-specific and do not include any specific technical considerations nor focus on task but rather on job level. That is, they are possibly not able to fully explain the changes in the world of work regarding the digitalization of tasks. As automation can foster and decrease the factor of job control, the influence of varying degrees of automation, moderated by perceived autonomy, and on workers' wellbeing and mental health might not be directly visible. As seen in the large-scale study on German workers (DiWaBe), this seems to be the case. More instructions by ICT were correlated with lower levels of perceived job control but did not influence mental health factors. Interestingly, more instructions by machines affected the feeling of control negatively and as predicted by the mentioned models, mental health. Therefore, models on the factor of job control can only partly explain the influence of AI on employees in actual working situations in the present survey. There are other factors such as task significance, feedback or social support that contribute to the overall working conditions and have not been included in the survey which could explain the missing link to mental health factors. The Job-Demand-Resources model by Demerouti et al., 2001 would support this assumption as it proposes that other interacting work conditions can function as resources, which have the ability to balance out demands, such as the decrease on job control. However, this would not account for the differences between instruction by ICT or machines. When looking at the models on LOAs that focus on the technical implementation of automation, a clear focus on task specific automation becomes apparent. They do not differentiate between different types of AI-based systems or sectors but rather dedicate attention to the specific human ability that is automated. According to these models, it is highly important which subtasks are automated in order to foresee the impact on workers. Both models assume that the automation of redundant tasks influences working conditions positively when the technology is reliable while taking away control from the human in tasks of expertise, has negative impacts. Overall, they only take away the decisional power from the human on the highest level, that is, under full automation. For all other levels, the human remains with a certain degree of decisional power. These models portray the optimal way of using automation in order to foster human performance while decreasing the negative effects it can have, such as a lower perceived level of job autonomy and control.

Results indicate that automation in occupational practice does not happen fully in line with this postulated model of automation. Instead, a substantial part of automation happens at the decision-making level, while executive actions remain with the human. The question remains why this process has led to significant effects on mental health factors when instructions came from machines, compared to instructions by ICT. According to all mentioned models, the reduction of perceived job control should have influenced mental health factors in both cases negatively if there are no other positive factors for workers that got instructions by ICT which would alleviate the impact of a reduced feeling of control. A possible explanation might be that work with ICT is accompanied with higher average levels of job control, so that a reduction by more instructions by technology does not lead to a critical level. This also emphasizes the application of the presented theories and models not on a broad overall level, but when considering the specific working task.

## Conclusion

Models and theories on human in control draw on well-established research in occupational psychology. In sum, literature has proven that less control and autonomy has negative effects on workers' job satisfaction, performance and mental health. These models clearly show the importance of the factor job control, as well as other factors, such as task significance, feedback and task variety. Due to more automation in the world of work and overall higher degrees of digitalization, automation technologies often take over different subtasks from humans. This happens on varying levels, sometimes leaving the human with supervisory tasks or simply following instructions. This transformation has led to the justified fear of loss of control in workers. Indeed, recent studies showed that a higher degree of automation can have detrimental effects such as loss of control, complacency, reduced situational awareness and task variety. Models on LOAs have therefore taken on the challenge to create an optimal pattern for task automation in which humans can remain in control while aided by technology to increase performance and optimize workload and the mentioned effects. However, they are very task specific and entail multiple loops to evaluate the degree to which the automation influences human performance. Unfortunately, they do not give specific guidelines for different tasks or sectors so that each task with a change in the degree of automation has to pass through the complete theoretical framework in order to have positive implications. The results of the DiWaBe study on German workers shows the large scope of digitalization as more than 90% of people are already working with ICT and nearly 50% with machines. These changes have made it important for stakeholders to highlight the principle of the **human being in control or preserving workers**' **autonomy** when designing AI-based systems (Rosen et al., 2022).

Although the assumed influence of a decrease in job control on mental health factors seen in the models by Ulich (2005), Karasek (1979) and Demerouti et al., 2001 as well as Hackman and Oldham (1975) cannot be seen consistently in the DiWaBe results, they are visible for workers who get more instructions by machines. This might be due to a higher average level of job control among (knowledge based) ICT-Work than machine work, preventing the demand-resources balance to reach critical levels. As literature emphasizes automation is a double-edged sword, it is crucial to closely monitor changes in automation from an objective point of view, taking productivity, reliability and profitability into account while also looking at automation from a worker's perspective in detail to face challenges for occupational health and safety. Furthermore, fostering positive work conditions such as good social support, feedback as well as opportunities for learning and personal development could provide a higher chance to turn automation into a resource (Demerouti et al., 2001; Demerouti, 2020). The technical models by Parasuraman et al. (2000) and Kaber and Endsley (1997) describe optimal ways when implementing automation, leaving control and supervisory subtasks with human while automating physical subtasks and information gathering.

Unfortunately, results indicate that automation in occupational practice does not happen in line with the models of optimal automation. Instead, there is a substantial level of decision-making by technology, which then exercises control on human employees. In addition, results show that this development is accompanied by a more unfavorable change in terms of demands and resources. Regarding the current rapid development of artificial intelligence, the possibilities to further automate decision-making within work processes will be increased massively, with the risk of more unfavorable working conditions. Therefore, it is of utmost importance from an occupational safety and health perspective to closely monitor and anticipate the implementation of AI in working systems. These results should then be considered continuously by policy making for workplace design, for example regarding in standardization procedures. The goal here is to avoid constellations where employees are too controlled by technology and are left with a high degree of demands and very limited resources. Instead, it would be favorable to use AI as an assistance tool for the employees, helping them to gather and process information and assisting them in decision-making.

## Data Availability Statement

The data analyzed in this study is subject to the following licenses/restrictions: It is planned on a medium-term to publish the data of the DiWaBe survey as weakly anonymized dataset within the Research Data Centre (FDZ) of the Federal Employment Agency (BA) at the Institute for Employment Research (IAB), Germany. Requests to access these datasets should be directed to MH, hartwig.matthias@baua.bund.de.

## Ethics Statement

Ethical review and approval was not required for the study on human participants in accordance with the local legislation and institutional requirements. Written informed consent for participation was not required for this study in accordance with the national legislation and the institutional requirements.

## Author Contributions

SW and PR conceptualized the paper's content and structure, and revised the manuscript. MH performed the data analysis. SN and MH wrote the first draft of the manuscript. All authors contributed to manuscript revision, read, and approved the submitted version.

## Funding

The DiWaBe survey was supported by the Interdisciplinary Social Policy Research Funding Network of the German Federal Ministry of Labour and Social Affairs.

## Conflict of Interest

The authors declare that the research was conducted in the absence of any commercial or financial relationships that could be construed as a potential conflict of interest.

## Publisher's Note

All claims expressed in this article are solely those of the authors and do not necessarily represent those of their affiliated organizations, or those of the publisher, the editors and the reviewers. Any product that may be evaluated in this article, or claim that may be made by its manufacturer, is not guaranteed or endorsed by the publisher.
